# Correlations Between Awareness of Illness (Insight) and History of Addiction in Heroin-Addicted Patients

**DOI:** 10.3389/fpsyt.2012.00061

**Published:** 2012-07-09

**Authors:** Angelo Giovanni Icro Maremmani, Luca Rovai, Fabio Rugani, Matteo Pacini, Francesco Lamanna, Silvia Bacciardi, Giulio Perugi, Joseph Deltito, Liliana Dell’Osso, Icro Maremmani

**Affiliations:** ^1^Vincent P. Dole Dual Diagnosis Unit, Department of Neurosciences, Santa Chiara University Hospital, University of PisaPisa, Italy; ^2^Association for the Application of Neuroscientific Knowledge to Social AimsLucca, Italy; ^3^SerT (Drug Addiction Unit)Pisa, Italy; ^4^G. De Lisio Institute of Behavioural SciencesPisa, Italy; ^5^Department of Psychiatry and Behavioural Science, New York Medical CollegeValhalla, NY, USA

**Keywords:** awareness of illness, compliance to treatment, heroin addiction, insight, mental status, natural history of addiction, psychiatry, severity of illness

## Abstract

In a group of 1066 heroin addicts, who were seeking treatment for opioid agonist treatment, we looked for differences in historical, demographic, and clinical characteristics, between patients with different levels of awareness of illness (insight). The results showed that, in the cohort studied, a majority of subjects lacked insight into their heroin-use behavior. Compared with the impaired-insight group, those who possessed insight into their illness showed significantly greater awareness of past social, somatic, and psychopathological impairments, and had a greater number of past treatment-seeking events for heroin addiction. In contrast with other psychiatric illnesses, the presence of awareness appears to be related to the passing of time and to the worsening of the illness. Methodologies to improve the insight of patients should, therefore, be targeted more directly on patients early in their history of heroin dependence, because the risk of lack of insight is greatest during this period.

## Introduction

Patients’ lack of insight into their mental illness is a complex and poorly investigated phenomenon which seriously influences psychosocial functioning, shows a clear correlation with the severity of psychiatric symptoms, and has been seen as related to treatment compliance, with important prognostic implications (Husted, 1999; Francis and Penn, 2001; Vender and Poloni, 2006).

In psychology and psychiatry, insight can mean the ability to recognize one’s own mental illness (Markova, 2005). Insight is also defined as awareness of mental illness. The interchangeability of these two terms derives directly from the definition of “insight,” and is widely supported by the current literature: in the validation process of several questionnaires designed to measure patients’ insight into mental illness, insight has been defined as “self-appraisal of Illness” (Marks et al., 2000), “judgment” (Kokoszka et al., 2008), “self-reflection on patients’ anomalous experiences” (Engh et al., 2007), “awareness of having a mental disorder” (Beck et al., 2004), and “awareness of psychiatric illness” (Roncone et al., 2003). Special attention has been paid to three different elements that are involved: insufficient awareness in patients of specific aspects of each form of the disease, the neurocognitive impairment entailed in each case, and consequent failures to comply with treatment.

An impaired level of insight has been suggested in psychiatry. With reference to mood disorders, patients’ awareness of illness has been shown to be related to the polarity of mood episode (poorer insight in mania if compared with mixed states and depression), and to the type of bipolar disorder (poorer insight in bipolar II stabilized patients, if compared with bipolar I stabilized patients; Pallanti et al., 1999; Dell’Osso et al., 2002; Varga et al., 2006). With reference to anxiety disorders, they have shown higher levels of insight if compared with mood disorders (Ghaemi et al., 2000). In psychotic disorders, a poor degree of insight has been associated with a poorer course of the illness and non-compliance with treatment that is necessary for patients (Husted, 1999). Unexpectedly, deficits in insight have not been found to be more common and severe in patients with schizophrenia than in those with bipolar disorder – a finding that has strong clinical, theoretical, and nosological implications (Pini et al., 2001). In the case of the obsessive-compulsive disorder, which is typically characterized by an excellent level of insight, a trend toward a lower level of insight has been noted in patients with somatic obsessions and in obsessive-bipolar patients (Marazziti et al., 2002). In post-traumatic stress disorder as well has been shown the need to improve awareness of illness to better understand the disease and the possible complications that it could occur (Khoury et al., 2010).

An impaired level of insight has been suggested and sometimes explored in Substance Use Disorders (SUD), too.

Substance abusers usually deny, or lack awareness of, their problem. Recent neuroscientific evidence suggests that the denial of problems related to drug use may be associated with alterations in various brain networks that contribute to insight. This integrated model includes the insula, which is involved in introception, self-awareness, and drug craving; the anterior cingulate, which is involved in behavioral monitoring and response selection; and the frontal and dorsal striatum, which is involved in automatic habit formation and self-awareness (Verdejo-Garcia and Perez-Garcia, 2008; Goldstein et al., 2009). Of particular interest is the role played by the insula in processes related to conscious introception, emotional experience, and decision-making. Within this framework the insula contributes to the way addicted individuals feel, remember, and decide about taking drugs – the main result being that drug addiction turns out to be like a naturally motivated behavior, closely comparable with eating and sex (Naqvi and Bechara, 2010).

As to alcohol, a close relationship has been observed between the state of patients’ insight and their level of motivation to change their own behavior (Jung et al., 2011). Some papers have pointed out the effect of insight on patients’ willingness to change their lifestyle, suggesting the need for brief insight-enhancement interventions addressed to patients with alcohol dependence (Kim et al., 2007). When a contemporary mental illness is present (in cases with dual diagnosis), this has been hypothesized to act as a fundamental factor in impairing insight level (Yen et al., 2009).

A few studies have been dedicated to investigating the role of insight in cocaine dependence. Some authors have examined the potential association between awareness of illness and drug-seeking behavior, and have gone on to propose, especially with cocaine subjects who are urine-negative, interventions to enhance insight, in order to improve their longer-term clinical outcomes (Moeller et al., 2010). Active cocaine abusers display a diminished neural response to errors, with particular reference to the anterior cingulate cortex, which is closely involved in error processing. The inability to detect or adjust performance in response to errors has been linked with clinical symptoms, including the loss of insight and perseverative behavior. According to this theory, behavioral deficits that probably contribute to the maintenance of drug dependence could derive directly from a cognitive dysfunction (Hester et al., 2007).

With reference to cannabis, there has been a great deal of debate over the role of THC in the pathogenesis of psychotic disorders; to date, cannabis use has been considered to be one of the most important risk factors for psychosis (Sevy et al., 2010; Hall and Degenhardt, 2011; Kuepper et al., 2011; Large et al., 2011). Whatever the cause, it is very difficult for many subjects to bring cannabis use to a stop during the early phases of recovery, not only because of prior habits of use, but also because of the attendant psychotic symptoms, including poor insight and judgment, lack of impulse control, and, most crucially, cognitive impairment. Moreover, most of these subjects are unable to grasp the fact that cannabis use is connected with the onset of symptoms. Of course, patients’ insight is a basic condition for understanding the need for treatment programs, and is still further damaged by on-going substance use (Miller et al., 2005).

In the field of heroin addiction, there is, in the literature, a worrying and unjustified shortage of data. This fact is even more disquieting when it is borne in mind that heroin addicts often fail to comply with methadone treatment when this is prescribed at appropriate blocking dosages. Lack of insight is a very difficult state for building the motivation that is indispensable for compliance or abstinence.

In our opinion medicine and, especially, addiction medicine can no longer be conceived as a simple intervention whose results depend exclusively on a physician’s decision. Patients have, in fact, become ever more closely involved in the therapeutic process, especially on rehabilitative grounds, so that they actually take part in their treatment, though not at a decision-making level (Falloon, 1988). Increasing efforts have been dedicated to informing and educating patients and significant ones about health issues, so making possible the active participation of any concerned citizen (Falloon, 1988, 1995; Falloon and Fadden, 1995). Thus, on rehabilitation and prevention grounds, it is crucial to provide information about the nature of the disease, its features and its course, while making clear which of the available treatments are the most effective (Anderson et al., 1986; Falloon, 1988; Bellack and Mueser, 1993), and overcoming misleading thinking styles.

We assume that a patient’s compliance with therapy is to some degree related to his/her insight. As a result, the assessment of insight is necessarily preliminary to an educational process that improves the patient’s active role in the therapeutic process. Correlations between the patient’s degree of insight and the history of addiction may be helpful to clinicians in identifying which patients are most in need of an educational intervention.

On the basis of these observations, a cohort study was designed with the aim of correlating the presence-absence of insight with the history of heroin addiction of patients entering opioid agonist treatment (AOT). It is, in fact, a key priority to be able to highlight the clinical features of patients without insight, because that ability makes it easy to select patients who need to be helped to develop their insights.

Study data were obtained from a general database of patients enrolled during the years 1994–2011 at the Vincent P. Dole Dual Diagnosis Unit, Santa Chiara University Hospital, Department of Psychiatry, University of Pisa, Italy. We selected patients whose awareness of illness data had already been obtained. This is, therefore, a retrospective, observational, cross-sectional study of heroin-dependent people at treatment entry.

## Materials and Methods

### Setting

The program in which our patients are placed after a starting evaluation, is characterized by a high threshold program. We believe that patients with better insight will best benefit from this type of program. All these considerations aroused the interest of our research group in the degree of insight of heroin-dependent patients as a feature to be included in any program that relies on opioid agonists. We believe we need to study patients’ insight before admission into methadone treatment (MMT). In our view stable methadone maintenance should be considered a good outcome. It is highly likely that a heroin user on stable MMT may think he/she has no problems and be given a score indicative of that user being “without insight” just because there is no problem in his/her life. We think that this bias may be “controlled,” by interviewing patients before treatment.

#### Subjects

The sample consisted of 1066 heroin-dependent patients (according to DSM III/IIIR/IV/IVR criteria); of these, 810 (76.0%) were males and 256 (24.0%) females. The average age was 29 ± 6 years old (range 16–51). Most of the patients were single (*N* = 681; 64.0%), had fewer than 8 years of education (*N* = 750; 70.5%), and were unemployed (*N* = 421; 39.5%).

Compared with females, males were older (*T* = 4.06; *p* = < 0.001) more often single (χ^2^ = 21.2; df 1; *p* = <0.001) and blue-collar workers (χ^2^ = 25.4; df 3; *p* < 0.001). By contrast, females were more often students and unemployed. No differences were observed as regards education, income, type of housing, place of birth or residence, or on the question of whether they were receiving public welfare benefits.

We selected patients that were coded 0 on the Drug Addiction History Questionnaire (DAH-Q, see below) for item 7 (classified as “without-insight patients,” *n* = 690). In the DAH-Q, the presence/absence of insight is related to the correctness of the patient’s knowledge about the nature of the disease with respect to its pathogenesis, prognosis, and treatment modalities (see Instruments). For our comparison group we selected patients coded 1 (partial or total insight, classified as “with-insight patients,” *n* = 376). These two groups were then compared for demographic, toxicological, psychopathological, and treatment-related variables.

All patients gave their informed consent to the anonymous use of their personal clinical data records for research purposes.

#### Instruments

##### Insight assessment

We started to collect data on awareness of illness of our heroin-dependent patients in 1994. We developed this interview on the basis of the different aspects that, in the literature, are considered to be involved in determining a patient’s level of insight. Several instruments in the last decade have been developed to allow the measurement of awareness of illness in psychiatric patients, but they have mainly been validated in psychotic and bipolar patients (Marks et al., 2000; Roncone et al., 2003; Beck et al., 2004; Markova, 2005; Engh et al., 2007; Camprubi et al., 2008; Kokoszka et al., 2008). Only one instrument, to the best of our knowledge, has been validated in alcoholic patients (Kim et al., 1998). Moreover, when we started to study insight in our patients, these instruments had not yet been worked out. Our clinical interview, therefore, has never been a structured interview. Our research group has, however, always interpreted the results of the interview in the same way, and has always encoded interview results in the same way at DAH-Q. This made possible the selection of patients without insight in a way incurring a low risk of false negatives.

Two psychiatrists who had almost 2 years of postgraduate experience carried out the interview. These two researchers had to agree on a patient’s lack of insight, and a senior researcher had to agree, too.

Subjects were asked

- Why they had applied for treatment, and what they would do if the substance were widely available at zero cost.
- What they think is required to bring about the termination of heroin use, what being addicted means, what “withdrawal” means, and why relapse often occurs after apparent remission.
- What they consider to be the most effective way of staying free of addiction, what objectives must be achieved for any treatment to be classified as effective, and about their views on special issues such as treatment in situations comprising hepatitis, and HIV infection.
- What kinds of professional skill are more or less important for treatments to be successful. The judgments expressed made it possible to understand indirectly whether subjects thought addiction belongs to the psychosocial or the biological sphere.

We considered patients to be without insight when all the following perceptions were present (code = 0):

- A patient denies having long-term problems with substance use control; he/she fails to understand the risk of relapsing after detoxification and/or tends to recognize a state of only partial well-being as amounting to the achievement of definitive healing;
- He/she is unable to relate the behavioral changes personally experienced to the chronic intoxication effects brought about by the substance;
- He/she considers agonist opioid treatment to be useless, and argues that their ability to recover from the disease is entirely due to their “willpower”.

On the other hand, we considered patients to be in possession of partial or total insight if one or more of the above situations were absent (code = 1). The results of this assessment were reported in DAH-Q items 7.

For these reasons, we cannot provide evidence of the psychometric properties of the instrument, nor of inter-rater reliability, because the instrument has changed slightly over the years. However, the fact that only two categories (patients without insight and patients with total or partial insight) were being compared may have reduced this bias. Control over bias from different raters was partially ensured by the supervision of a senior psychiatrist (I. Maremmani), who continued to work at the Psychiatric Clinic of the University of Pisa for as long as the research lasted and still works there.

##### Drug addiction history questionnaire

The DAH-Q (Maremmani and Castrogiovanni, 1989) is a multi-dimensional questionnaire that comprises the following eight areas: 1 – demographic data, 2 – physical health, 3 – mental status, 4 – social adjustment and environmental factors, 5 – substances abused, 6 – substance abuse modalities (heroin intake, modality of use, stages of illness, nosography), 7 – treatment history, and 8 – addiction history (age at first contact, age at onset of continuous use, dependence length, and age at first treatment). The questionnaire rates 10 presence-absence items: 1 – somatic comorbidities, 2 – abnormal mental status, 3 – work problems, 4 – household problems, 5 – sexual problems, 6 – socialization and leisure time problems, 7 – drug-related legal problems, 8 – poly-substance abuse, 9 – previous treatment, 10 – combined treatments.

We encoded the modality of use as follows: 1 – stables, 2 – junkies, 3 – two worlders, 4 – loners according to Lahmeyer’s classification (Lahmeyer et al., 1988). “Stables” are opioid addicts who espouse conventional values, hold legitimate jobs, are generally law-abiding and do not associate with other addicts. “Hustlers,” otherwise called “junkies” or “criminal addicts,” are closely identified with an addict subculture, are not legitimately employed, and subsist on the proceeds of criminal activities. “Two-worlder” addicts engage in criminal activities and associate with other addicts, but are also legitimately employed. “Loner” addicts are not involved either in the addict subculture or the conventional culture. They are usually unemployed, and live on welfare benefits rather than on the proceeds of criminal activities. These uninvolved addicts may have severe psychological disorders.

Their development of addiction may be considered to consist of three stages: 1 – acute (immediate) drug effects (Honeymoon Stage); 2 – transition from recreational use to patterns of use consistent with addiction (Increasing Dose Stage); and 3 – end-stage addiction, which is characterized by an overwhelming desire to obtain the drug, a diminished ability to control drug-seeking and reduced pleasure from biological rewards (Revolving Door Stage; Kalivas and Volkow, 2005).

Considering the clinical typology, drug addicts can be divided into 1 – reactive (presence of psychosocial stressors before using heroin), 2 – self-therapeutic (presence of psychiatric stressors before using heroin), and 3 – metabolic (no psychosocial or psychiatric antecedents; Maremmani and Popovic, 2009).

##### Psychiatric diagnostic evaluation

Psychiatric disorders were investigated on the basis of the DSM-IV Decision Trees for Differential Diagnosis (APA, 2000). Each decision tree starts with a set of clinical features. When one of these features is a prominent item of the current clinical picture, the clinician will ask a series of questions to rule in or rule out a number of disorders. The questions are just approximations to the diagnostic criteria that are used, and are not meant to replace them. Three decision trees have been used: “Differential Diagnosis of Psychotic Disorders” (initial clinical features: delusions, hallucinations, disorganized speech, or grossly disorganized behavior); “Differential Diagnosis of Mood Disorders” (initial clinical features: depressed, elevated, expansive, or irritable mood; two separate items record the presence of depression and/or any tendency toward the bipolar spectrum as testified by an elevated, expansive, or irritable mood); “Differential Diagnosis of Anxiety Disorders” (initial clinical features: symptoms of anxiety, fear, avoidance, or increased arousal).

As to bipolar spectrum diagnoses, histories of previous hypomanic episodes, as well as temperamental characteristics, were explored using the criteria listed in the SID, the Semi-structured Interview for Depression (Cassano et al., 1989). All information was gathered from the patient and at least one close relative (usually from parents or siblings); in addition, all available clinical records were carefully examined. Inquiries into temperamental attributes were made about the habitual behavior of the patient – during periods free of affective episodes – by gathering information from the patient and significant others. Although it may seem strange that such figures have been documented for addicted patients, Italian addicts find it hard to become detached from their families, despite the disruption of family relationships. In fact, almost 90% of the patients in our sample were still living with their original or acquired families.

Our operational criteria for affective temperaments have been drawn from the University of Tennessee (Akiskal and Mallya, 1987) modification of the Schneiderian descriptions (Schneider, 1958). The SID, developed as part of the Pisa-Memphis (now San Diego) collaborative study on affective disorders, has been used with over 2000 patients at the time of writing; its reliability for diagnostic assessment of patients and their temperaments has been documented elsewhere (Perugi et al., 1990, 1997). The SID was resorted to with the aim of raising the level of diagnostic accuracy with respect to bipolar disorders. On the hypothesis that minor bipolar syndromes were overrated, that bias would not affect the rate of DD. In fact, the relationship between outcome and specific diagnostic subgroups is beyond the study’s terms of reference.

Patients were evaluated while outside acute phases, for which hospitalization would often be required, so as to reduce the diagnostic ambiguity between intoxication-related symptoms and spontaneous mental disorders. In cases where further information emerged on clinical grounds or from later interviewing, diagnoses were reviewed.

When an independent psychiatric disorder is concomitant with a substance abuse disorder, we consider the patient as being affected by a dual diagnosis condition.

#### Data analysis

The two groups selected on the basis of the DAH-RS item 7 score for the presence/absence of insight were compared for the demographic and addiction history of each group by means of the chi-square test for categorical variables, and Student’s *t*-test for continuous variables. Variables for which a statistically significant difference between the two groups was detected (*p* < 0.05) were treated (inserted) as independent variables in a logistic regression analysis with the presence/absence of insight as a binary outcome.

Multiple regression is a general statistical technique through which one can analyze the relationship between a dependent or criterion variable and a set of independent or predictor variables. Multiple regression may be viewed as a descriptive tool that can “simplify” the prediction equation by deleting independent variables that do not add substantially to prediction accuracy, once certain other independent variables are included.

All analyses were carried out using the statistical package of SPSS (version 20.0). Since this is an exploratory study, statistical tests were considered significant at the *p* < 0.05 level.

## Results

### Demographic data

With respect to demographic data, no differences were observed regarding age, gender, marital status (single or otherwise), occupation (student, white-collar, blue-collar, and unemployed), income (poor or better off), and living situation (alone or otherwise). Heroin addicts with and without insight significantly differed as regards education: 66.7% of with-insight heroin addicts had had over 8 years of education, whereas 72.6% of those without insight had had fewer than 8 years of education (χ^2^ = 4.06, *p* = 0.043).

### DAH-Q factors and addiction history

Table 1 shows differences between heroin addicts with and without insight regarding DAH-Q factors. No differences were observed among work, social, and leisure problems. Compared with heroin addicts without insight, with-insight heroin addicts frequently showed more somatic and psychopathological comorbidity, household major problems (parental relationships, marital/partner relationship, and parental role), problems in love relationships, legal problems, poly-abuse, and past unsuccessful treatments. In addition to heroin, they also abuse a greater number of substances and had received many different treatments previously. In summary, the clinical picture of drug addiction observed was more severe in heroin addicts with than without insight.

**Table 1:** **DAH-RS factors and addiction history**.

	*N* valid	With insight *N* = 376	Without insight *N* = 690	Chi	*p*
		*N* (%)	*N* (%)	
Somatic comorbidity	1065	280 (74.5)	460 (66.8)	6.81	0.009
Work major problems	1022	234 (63.4)	415 (63.6)	0.00	0.964
Household major problems	1014	311 (83.4)	428 (66.2)	35.17	<0.001
Parental relationships	1002	245 (67.1)	363 (57.0)	9.99	0.001
Marital/partner relationships	282	169 (68.7)	253 (51.6)	19.50	<0.001
Parental role	402	87 (84.5)	126 (42.1)	55.09	<0.001
Loving major problems	1026	225 (63.7)	356 (54.3)	8.42	0.003
Social and leisure major problems	1055	172 (46.4)	307 (45.2)	0.12	0.721
Legal problems	1057	168 (45.0)	210 (30.7)	21.60	<0.001
Poly-abuse	1062	269 (71.5)	318 (46.2)	63.38	<0.001
First treatment	1066	104 (27.9)	260 (37.8)	10.64	0.001
		*M* ± SD	*M* ± SD	*T*	*p*
No. somatic comorbidities	1065	1.67 ± 1.5	1.33 ± 1.3	−3.61*	<0.001
No. psychiatric comorbidities	1066	4.15 ± 2.5	1.73 ± 2.1	−15.80*	<0.001
No. abused substances	1062	3.61 ± 1.5	2.53 ± 1.7	−10.25	<0.001
No. different treatments in past	1066	1.79 ± 1.3	1.39 ± 1.5	−5.14*	<0.001
Age at first contact (years)	991	18 ± 4	19 ± 4	0.96	0.336
Age at onset of continuous use	979	21 ± 5	21 ± 5	−0.38	0.703
Dependence length (in months)	895	89 ± 68	104 ± 90	−2.28*	0.022
Age at first treatment	973	25 ± 5	26 ± 6	2.21	0.027

*Mann–Whitney *U*–Wilcoxon rank sum *W* test.

Considering variables characterizing drug addiction history, no differences were found regarding their age of first consumption and their age of continuous use of heroin. The dependency length of with-insight patients was shorter and their age at first treatment younger.

### Mental status at treatment entry and diagnosis

Regarding patients’ mental status at treatment entry, only the presence/absence of abnormal consciousness and delusions revealed no significant differences between the two groups evaluated for insight. 22.3% of with-insight patients showed memory alterations, as opposed to 7.7% of without-insight patients (χ^2^ = 46.20, *p* < 0.001). 54.3% of with-insight patients showed anxiety, against 33.5% of those without insight (χ^2^ = 43.20, *p* < 0.001). 61.1% of with-insight patients showed a major depressive episode, against 38.4% of those without insight (χ^2^ = 46.20, *p* < 0.001). 49.2% of with-insight patients showed sleep disturbances, against 27.1% of those without insight (χ^2^ = 52.08, *p* < 0.001). 25.0% of with-insight patients showed eating disturbances, as opposed to 11.2% of those without insight (χ^2^ = 34.49, *p* < 0.001). 34.7 and 30.8% of with-insight patients were excited and violent, against 18.6 and 16.3% of without-insight ones, respectively (χ^2^ = 34.22 and 30.32, *p* < 0.001). 14.8% of with-insight patients reported suicidal ideation, as opposed to 6.0% of those without insight (χ^2^ = 22.75, *p* < 0.001). 7.5% of with-insight patients showed hallucinations, against 3.1% of those without insight (χ^2^ = 10.85, *p* < 0.001). In summary, patients with-insight more frequently show an abnormal mental status upon entering treatment.

Table 2 shows differences between heroin addicts with and without insight as regards diagnoses. With-insight patients were more frequently diagnosed as dual diagnosis patients. In particular, they were diagnosed as having recurrent depression and bipolar spectrum disorders.

**Table 2:** **Diagnoses**.

	*N* valid	With insight *N* = 376	Without insight *N* = 690	Chi (df)	*p*
		*N* (%)	*N* (%)	
With psychiatric comorbidity	569				
Chronic psychosis		40 (10.6)	49 (7.1)		
Depression, recurrent		107 (28.5)*	154 (22.3)*		
Bipolar spectrum		81 (21.5)*	64 (9.3)*		
Anxiety disorders		27 (7.2)	47 (6.8)		
Without psychiatric comorbidity	497	121 (32.2)*	376 (54.4)*	60.35 (3)	<0.001
Dual diagnosis	1066	255 (67.8)	314 (45.5)	48.68 (1)	<0.001

*p < 0.05.

### Lifetime reported concomitant abuse of substances

With respect to lifetime self-reported concomitant substance abuse at treatment entry, with-insight patients used more alcohol frequently than without-insight ones (41.8 vs. 30.2%, χ^2^ = 14.43, *p* < 0.001), CNS depressants (36.2 vs. 23.3%, χ^2^ = 19.92, *p* < 0.001), CNS stimulants (60.4 vs. 36.7%, χ^2^ = 54.93), hallucinogens (41.1 vs. 21.8%, χ^2^ = 44.09, *p* < 0.001), cannabinoids (79.0 vs. 54.3%, χ^2^ = 63.48, *p* < 0.001), illegal methadone (36.3 vs. 18.7%, χ^2^ = 39.70, *p* < 0.001). Only the concomitant use of inhalants revealed no significant differences.

### Heroin (AB)use modalities

Table 3 shows the differences between heroin addicts with and without insight regarding their abuse modalities. Heroin addicts with-insight more frequently demonstrated the following characteristics: multi-daily heroin intake, junkie and two worlder modalities of heroin use located in the intermediate or “dose-increasing” phase of illness, having psychiatric antecedents before heroin use and having been treated previously without success. They were more frequently diagnosed as dual diagnosis patients. Conversely, without-insight patients showed the following characteristics: daily heroin use, stable modality of use, being in the “revolving door” phase of illness, having no psychosocial or psychiatric antecedents prior to heroin use.

**Table 3:** **Abuse modalities**.

	*N* valid	With insight *N* = 376	Without insight *N* = 690	Chi	*p*
		*N* (%)	*N* (%)	
**Heroin intake**	965				
Sporadic		44 (12.5)	90 (14.7)		
Weekly		10 (2.8)	19 (3.1)		
Multi-weekly		41 (11.6)*	31 (5.1)*		
Daily		81 (22.9)*	273 (44.6)*		
Multi-daily		177 (50.1)*	199 (32.5)*	60.21	<0.001
**Modality of use**	990				
Stables		164 (45.3)*	402 (64.0)*		
Junkies		110 (30.4)*	116 (18.5)*		
Two worlders		72 (19.9)*	71 (11.3)*		
Loners		16 (4.4)	39 (6.2)	41.37	<0.001
**Periodic self-detoxification**	950	263 (74.9)	463 (77.2)	0.61	0.433
**Stages of heroin addiction**	946				
Encounter or honeymoon		29 (8.1)	44 (7.5)		
Intermediate or dose-increasing		94 (26.4)*	96 (16.3)*		
Repeated detoxification or revolving door		233 (65.4)*	450 (76.3)*	15.08	<0.001
**Clinical typology**	935				
Reactive (psychosocial antecedents)		43 (12.3)	70 (12.0)		
Self-therapeutic (psychiatric antecedents)		86 (24.6)*	106 (18.1)*		
Metabolic (no psychosocial or psychiatric antecedents)		221 (63.1)*	409 (69.9)*	5.94	0.051
**Past treatments**	1060	269 (72.1)	427 (62.2)	10.64	0.001

*p < 0.05.

### Previous and concomitant treatments

With respect to previous treatments the with-insight patients have been treated more frequently with short-term methadone than without-insight ones (46.5 vs. 30.5%, χ^2^ = 26.86, *p* < 0.0001). The same occurred for psychotherapy (26.2 vs. 17.3%, χ^2^ = 11.84, *p* < 0.0001) and psychopharmacology (31.5 vs. 25.6%, χ^2^ = 4.18, *p* = 0.040). With-insight patients have also been treated more frequently in a therapeutic community (32.4 vs. 26.0%, χ^2^ = 4.84, *p* = 0.027). No differences between groups were observed regarding methadone and naltrexone maintenance treatment. With respect to concomitant treatments, with-insight patients received more psychopharmacotherapy (26.4 vs. 16.2%, χ^2^ = 15.8, *p* < 0.001). No differences were observed as regards psychotherapy or therapeutic community interventions.

### Predictors of insight

Table 4 shows the results of the backward logistic regression including as predictive factors those that proved to be significantly correlated with the presence/absence of insight in heroin addicts in the univariate analysis. On the basis of this analysis, the odds of having no insight were higher for patients with daily heroin intake (OR 1.57), with absence of poly-abuse (OR 1.79) and with absence of dual diagnosis (OR 1.75). Conversely, the odds of having insight were higher for multi-weekly heroin intake (OR 0.55), junkies (OR 0.42) or two worlders (OR 0.37), heroin addicts, and heroin addicts with a satisfactory household situation (OR 0.56).

**Table 4:** **Predictors of non-insight**.

Predictors	*B*	Exp (*B*)	−95% CI	+95% CI	*p*
Heroin intake					0.004
Multi-weekly	−0.58	0.55	0.32	0.96	0.036
Daily	0.45	1.57	1.10	2.25	0.013
Modality of use					0.001
Junkies	−0.85	0.42	0.20	0.87	0.020
Two worlders	−0.96	0.37	0.18	0.79	0,010
Poly-abuse: absence	0.58	1.79	1.33	2.42	0.000
Household: satisfactory situation	−0.56	0.56	0.42	0.76	0.000
Dual diagnosis: absence	0.56	1.75	1.29	2.37	0.000

*Backward logistic regression. Statistics: χ^2^ = 118.57, df 10, *p* = 0.000*.

## Discussion

In our sample, with-insight heroin addicts present a higher level of education, appear to show greater severity from a clinical point of view, present a shorter dependence length and are younger at first treatment. The presence of insight is associated with impaired mental status and with a stronger record of lifetime concomitant substance abuse at treatment entry. Insight is also associated with abuse modalities such as being junkies or two worlders with a multi-daily and multi-weekly heroin intake. With-insight patients more frequently than those without insight report previous short-term methadone treatments, psychotherapy, a concomitant psychopharmacology treatment, and a previous therapeutic community experience. As to diagnosis, with-insight patients are more frequently dual diagnosis depressed patients belonging to a bipolar spectrum. In our sample, being two worlders or junkies, presenting household major problems, and being dual diagnosis poly-abusers with a multi-weekly heroin intake, seem to correspond to the highest likelihood of belonging to the with-insight group of patients.

On the basis of our data the awareness of illness often appears to be absent in heroin addicts, as happens in a wide range of psychiatric conditions, too, including mood disorders, psychotic disorders, and the obsessive-compulsive disorder with a prevalence of somatic obsessions (Husted, 1999; Pallanti et al., 1999; Pini et al., 2001; Dell’Osso et al., 2002; Marazziti et al., 2002; Varga et al., 2006). An impaired level of insight has likewise been reported in the literature among other substance abusers (alcohol, Kim et al., 2007; Yen et al., 2009; cocaine, Hester et al., 2007; Moeller et al., 2010; and cannabis, Miller et al., 2005), in a pattern displaying similarities and some important differences.

In our sample there is a correlation between insight and a patient’s educational level. This relationship, to our knowledge, had been poorly investigated, and is thus difficult to discuss thoroughly. Despite this, as far as our heroin-dependent patients are concerned, education seems to promote insight. One very simple explanation may be that the impaired insight group, who achieved a lower level of education, might simply have been unaware that addiction is a disease and are thus led to believe in willpower by their lack of formal knowledge. This finding strengthens the idea that we need to improve the counseling provided to our heroin addict patients.

From a clinical point of view, with-insight heroin addicts belonging to our sample appear to display greater severity. Among alcohol-dependent patients the state of patients’ insight has been correlated with their level of motivation to change their lifestyle (Kim et al., 2007; Jung et al., 2011); if that finding is correct, the same kind of motivation could arise in heroin addicts from a painful and distressing lifestyle marked by somatic comorbidity, household, loving and legal problems, poly-abuse, and unsuccessful treatments in the past. Especially multiple substance abuse is usually correlated with a low insight (Martinotti et al., 2009).

The presence of insight in our heroin addict group was also associated with impaired mental status, psychopathological comorbidity, and the presence of a dual diagnosis, with a prominent role played by bipolar spectrum and recurrent depression. These observations are in open contrast with the literature, as the presence of a concomitant mental illness has been hypothesized to be a fundamental factor impairing insight level, both in substance abuse (alcohol and cannabis; Miller et al., 2005; Yen et al., 2009), and psychiatric disorders (Husted, 1999; Francis and Penn, 2001; Vender and Poloni, 2006). In even greater contrast with the literature is the evidence of a correlation between a preserved awareness of illness and the datum of belonging to a bipolar spectrum: a greater relative impairment of insight has, in fact, been observed in mood vs. anxiety disorders (Ghaemi et al., 2000). Moreover, if we consider the obsessive-compulsive disorder, which is marked out by good levels of insight in patients, a trend toward a lower level of insight has been noted in obsessive-bipolar patients with a history of repeated manic or hypomanic episodes (Marazziti et al., 2002). In reality, this observed correlation between insight and bipolarity cannot be considered surprising, when we consider that bipolar-I patients show higher levels of insight than bipolar-2 patients (Pallanti et al., 1999). There is no doubt that bipolar 1 illness is more severe than bipolar 2, as is demonstrated by its acute consequences, such as psychosis and hospitalization. Likewise, bipolar heroin addicts are patients whose condition is more severe than uncomplicated heroin-dependent ones. Thus the correlation between severity of illness and insight appears to be widely supported. Considering bipolar disorders and substance abuse from a unitary perspective, we propose the following hypothesis: as to mood disorders, insight is absent in pure mania, which, despite its dysfunctional aspects, is usually experienced by patients as a pleasurable and rewarding experience; among heroin addicts, insight seems to be impaired whenever heroin is enjoyed without experiencing its destructive consequences on a social or clinical plane. Insight into an experience might prove to be related to the polarity of affect that is related to this experience (Cocores et al., 1987; Maremmani et al., 2002, 2006, 2007a, 2009; Perugi et al., 2002; Brousse et al., 2008; Maremmani, 2008; Pacini et al., 2009; Pani et al., 2010).

In general, the level of patients’ insight has been reported to be related to compliance with treatments and, as a result, with retention in treatment (Husted, 1999; Kao and Liu, 2010; Segarra et al., 2010). Our results are consistent with a previous study by us, which compared the retention in treatment of opioid-dependent subjects with and without Axis-I psychiatric comorbidity. The absence of differences between the two groups of patients in terms of retention in treatment, despite the higher methadone dose required for clinical stabilization of dual diagnosis patients, support the present observations indicating that a concomitant Axis-I disorder is not responsible for lower levels of insight (Maremmani et al., 2000). To sum up, among heroin addicts Axis-I comorbidity turns out to play an opposite role, by comparison with other psychiatric conditions, on levels of insight and retention rates. Even if further studies are needed, we can speculate that the positive impact of psychiatric comorbidity on insight levels might be mediated by the therapeutic effect of methadone treatment on the psychopathological symptoms of psychiatric patients, and by the consequent eventual improvement of their quality of life. These observations are widely supported by the evidence in favor of the antidepressant, anti-manic, stabilizing, antipsychotic, and anti-panic effects of long-acting opioids (Spensley, 1976; Berger et al., 1980; Gerner et al., 1980; Clouet, 1982; Exstein et al., 1982; Gold et al., 1982; Varga et al., 1982; Emrich, 1984; Bodkin et al., 1995; Levinson et al., 1995; Callaway, 1996; Krausz et al., 1996; Maremmani et al., 1998, 2000; Pani et al., 1999).

In with-insight heroin addicts, at their treatment entry, stronger lifetime concomitant substance abuse has been reported. This observation is consistent not only with the evidence in favor of greater severity in this kind of patient (Maremmani et al., 2007b), but also with the higher rate of previous unsuccessful treatments (especially short-term methadone treatments) that has been recorded. Craving for alcohol and cocaine has, in fact, been found to be correlated with low or non-blocking methadone dosages in methadone-maintained patients (Stine et al., 1992; Tennant and Shannon, 1995; Foltin and Fischman, 1996; Lubrano et al., 2002).

The fact that abuse modalities such as being junkies or two worlders with a multi-daily and multi-weekly heroin intake have also shown a positive correlation with a patient’s level of insight is consistent with the trend observed for other clinical features related to severity of illness. Stable heroin addicts, on the other hand, might be expected to present low levels of insight by virtue of their being more socially integrated, and exempt from any of the major negative consequences of heroin addiction.

On the topic of therapeutic interventions, previous short-term methadone treatments, psychotherapy, a concomitant psychopharmacology treatment, and a previous therapeutic community experience are features that have been reported in with-insight heroin addicts. A history of unsuccessful treatments may act as a factor promoting the development of higher levels of insight. If patients are incapable of breaking away from heroin despite various attempts to do so, they may well start to suspect that success is not only a matter of being armed with “good intentions.”

### Clinical implications

Looking at the addiction history of our patients, we could say that the level of insight seems to improve lately, with the progression of the illness. In clinical practice it is frequent to have heroin addicts at the earlier stages of their disease that did not want to start long-acting opioid treatment, not only because they did not believe to have a problem related to substance abuse, but also because they are convinced that the choice to quit with heroin is merely based on their “willpower”. On the other hand, it is also frequent to observe heroin addicts that came to clinical setting looking for any kind of help when they are in the last stages of addiction. In these conditions, heroin addicts, who, like in our sample, luckily present a better awareness of illness, are frequently junkies or two worlders with multi-daily heroin intake, present previously unsuccessful treatments and have a dual diagnosis, presenting a mental illness, frequently belonged to the bipolar spectrum, that could be related to their addiction history (Maremmani et al., 2011). In other words insight came in the end and its presence correlate with a more severe clinical presentation. Thus, clinicians have the duty to improve the motivation to treatment in heroin addicts, especially in the first phases of addiction in which patients still did not show insight of illness and present a less severe clinical presentation.

### Limitations

Obvious limitations on this study include the fact that we are reporting on a retrospective analysis carried out on a large cohort of patients; it was not a study specifically designed to elucidate this issue. The division of patients in to low and high insight groups was based on their perceptions of the importance and severity of their opioid abuse. Generally speaking, those with greater insight were more attuned to the importance of their illness, yet it may also be fair to simply hypothesize that the more severe an illness is, the more likely someone is to develop an awareness of its importance. This may not, however, amount to any precise achievement of “insight” in the usual psychological sense. In addition our tool may not really measure insight but might show the awareness of negative life situations.

In the literature, drug abuse and other psychiatric conditions have been associated with a fall in the neural response to errors, in those cortical regions that are thought to be critical to error processing. This cognitive dysfunction has the potential to contribute to the persistence of drug abuse, but has not been adequately targeted by our evaluation of these patients’ level of insight into their illness (Hester et al., 2009). Some measure of insight as a psychological variable independent of patients’ awareness of how severe of their disease was would have strengthened this study. In the futur**e**, an assessment of this kind may be employed in identifying the high vs. low insight groups.

## Conclusion

In conclusion, the awareness of illness often appears to be absent in heroin addicts, as this situation can be found in a wide range of psychiatric conditions. Compared with the impaired-insight patients, those who possessed insight into their heroin abuse behavior had gone further in their addictive career, showed significantly greater awareness of past social, somatic, and psychopathological impairments, and had experienced a greater number of past treatment-seeking events for heroin addiction. In contrast with other psychiatric illnesses, the presence of awareness appears to be related to the passing of time and the worsening of the illness. From a clinical point of view, methodologies to improve the insight of patients should, therefore, be targeted more directly on patients early in their history of heroin dependence, because the risk of lack of insight is greatest during this period.

## Author Contributions

Angelo Giovanni Icro Maremmani, Luca Rovai, Matteo Pacini, Fabio Rugani, and Icro Maremmani planned the study, participated in its design and coordination, and helped to draft the manuscript. Francesco Lamanna, Silvia Bacciardi, Giulio Perugi, Joseph Deltito, and Liliana Dell’Osso revised the literature and participated in the interpretation of data. All the authors read and approved the final manuscript.

## Conflict of Interest Statement

The authors declare that the research was conducted in the absence of any commercial or financial relationships that could be construed as a potential conflict of interest.
